# The Impact of Obesity on Influenza Vaccine Immunogenicity and Antibody Transfer to the Infant During Pregnancy

**DOI:** 10.3390/vaccines12121307

**Published:** 2024-11-22

**Authors:** Michelle Clarke, Suja M. Mathew, Lynne C. Giles, Ian G. Barr, Peter C. Richmond, Helen S. Marshall

**Affiliations:** 1 Women’s and Children’s Health Network, North Adelaide, SA 5006, Australia; michelle.clarke@adelaide.edu.au (M.C.); suja.mathew@adelaide.edu.au (S.M.M.); 2 The Robinson Research Institute, Adelaide Medical School, The University of Adelaide, Adelaide, SA 5005, Australia; 3 School of Public Health, The University of Adelaide, Adelaide, SA 5005, Australia; lynne.giles@adelaide.edu.au; 4 WHO Collaborating Centre for Reference and Research on Influenza, The Peter Doherty Institute, Melbourne, VIC 3000, Australia; ian.barr@influenzacentre.org; 5 Discipline of Pediatrics, University of Western Australia, Perth, WA 6009, Australia; peter.richmond@uwa.edu.au; 6 Wesfarmers Centre of Vaccines and Infectious Diseases, Kids Research Institute of Australia, Perth Children’s Hospital, Perth, WA 6009, Australia

**Keywords:** influenza, vaccine, obesity, pregnancy

## Abstract

Background/Objectives: Influenza vaccination is recommended for pregnant women, offering the dual benefit of protecting pregnant women and their newborn infants against influenza. This study aimed to investigate the impact of body mass index (BMI) on influenza vaccine responses in pregnant women and their newborns. Methods: Participants included pregnant women attending the Women’s and Children’s Hospital in South Australia between 2018 and 2021. Maternal blood samples were collected prior to and at 1 and 6 months post-influenza vaccination to measure antibody responses by hemagglutination inhibition (HI) assay. Cord blood samples were also collected. The percentages of participants achieving HI titre ≥40 were compared between obese and non-obese groups. Results: A total of 73 women were enrolled and received quadrivalent influenza vaccination at a mean age of 32 years (range 21–44 y) and median gestation of 24 weeks (range 11–37 weeks). BMI at vaccination was ≥30 kg/m^2^ for 21/73 women (29%). Most pregnant women demonstrated antibody titres ≥ 40 to all four influenza vaccine strains at 1 month post-vaccination regardless of BMI category (BMI ≥ 30 kg/m^2^: 19/20; 95% vs. BMI < 30 kg/m^2^: 47/49; 96%). At 6 months post-vaccination, 12/17 (71%) obese women compared to 36/43 (84%) non-obese women (*p* = 0.25) maintained HI titres ≥ 40. Cord blood serology showed HI titres ≥ 40 for 11/17 (65%) infants born to mothers with BMI ≥ 30 compared to 30/35 (86%) infants delivered by mothers with BMI < 30 kg/m^2^. Conclusions: A high BMI did not impair influenza vaccine antibody responses in pregnant women at 1 month post-vaccination. However, at 6 months post-vaccination, and in the cord blood samples, the percentages maintaining HI titre ≥ 40 were lower for obese women than for non-obese pregnant women.

## 1. Introduction

Influenza is a highly contagious respiratory viral disease that can affect people of all ages worldwide. Whilst seasonal variations are common, it is estimated that one in five children and one in ten adults are infected with influenza annually [1]. Although most people recover without requiring medical attention, pregnant women, very young infants and people with obesity are some of the risk groups that are particularly vulnerable to more severe illness following influenza infection [2,3,4,5,6,7,8]. In Australia, and many other countries, influenza vaccination is recommended during pregnancy for the dual benefit of protecting mothers and their infants from severe influenza illness and its complications [8,9,10]. Given that influenza vaccines are only licenced for infants from 6 months of age, maternal influenza vaccination during pregnancy is currently the best strategy available for protecting young infants. Infants are at risk of complications from influenza infection and have the highest age-specific influenza hospitalisation rates [8,11,12]. Young infants therefore rely on passive antibody transference from their mother during pregnancy to provide protection against influenza infection.

Pregnant women are also at greater risk of more severe influenza illness than non-pregnant women [4,6]. Furthermore, obesity is recognised both as a risk factor for more severe influenza infection [2,3,7,13] and as a potential mediator of impaired vaccine responses [14,15,16,17,18,19]. To date, very few studies have examined the association between obesity and influenza vaccine responses in pregnant women [20,21], although a study in non-pregnant adults found that obesity was associated with a more rapid decline in antibodies at 12 months post trivalent influenza vaccination [17]. There are no published studies comparing influenza antibody titres in cord blood in regard to obese vs. non-obese women receiving influenza vaccination during pregnancy. More than 30% of Australian females are living with obesity at present [22]. Globally, in 2022, an estimated 16% of adults are living with obesity; however, this varies greatly by region, with lower rates of adult females living with obesity being seen in countries such as Vietnam, Japan and Ethiopia (<5%) and rates higher than 30% being seen in countries such as USA (43%), South Africa (46%) and New Zealand (35%) [23], Therefore, understanding whether obese pregnant women can develop adequate influenza antibody vaccine responses, and maintain and transfer adequate influenza-specific antibodies to their infants, is important for ensuring that the current vaccine programme is providing optimal protection for pregnant women and their young infants irrespective of their BMI.

## 2. Materials and Methods

### 2.1. Study Procedures and Participants

This prospective cohort study was conducted at a tertiary maternity and paediatric hospital in South Australia between August 2018 and May 2022. Pregnant women attending antenatal services were approached by research nurses and invited to participate if they met the study eligibility criteria. From May 2020, printed flyers summarising the study with an opportunity to register interest were provided in antenatal packs given to women attending the antenatal services, as face-to-face recruitment was restricted due to the COVID-19 pandemic. Inclusion criteria required participants to be at least 18 years of age, have a sufficient enough understanding of the English language to provide valid consent and to be willing to receive a seasonal influenza vaccination. Women with significant medical conditions, including insulin-dependent diabetes, immunosuppressive conditions or requiring immunosuppressive medication, were excluded. In addition, women with any contraindication to vaccination, or those who had received any immunoglobulin therapy, blood or blood products or influenza vaccination within the previous six months, were excluded.

After written informed consent, pregnant women were administered a 0.5 mL dose of a licenced, seasonal quadrivalent inactivated influenza vaccine (Fluquadri^®^, Sanofi-aventis, Macquarie Park, Australia). If the participant was also eligible for a dTpa vaccine (gestation > 20 weeks) and had not already received a pertussis-containing vaccine during their current pregnancy, a dose of Adacel^®^ (Sanofi-aventis, Macquarie Park, Australia) was also administered. Vaccines were administered by Immunisation Nurses or medical officers according to the Australian Immunisation Handbook recommendations. Participants’ heights and first-trimester weights were assessed by a study investigator at the first study visit. BMI was used to classify participants as either obese (≥30 kg/m^2^) or non-obese (<30 kg/m^2^). Clinical and demographic information, including maternal age, country of birth, gestational weeks at vaccination, prior influenza vaccination history and laboratory-confirmed respiratory illnesses within the previous 12 months, were also recorded. Serious adverse events were collected during the study via participant diaries and a review of medical records following study completion.

**Figure 1:**
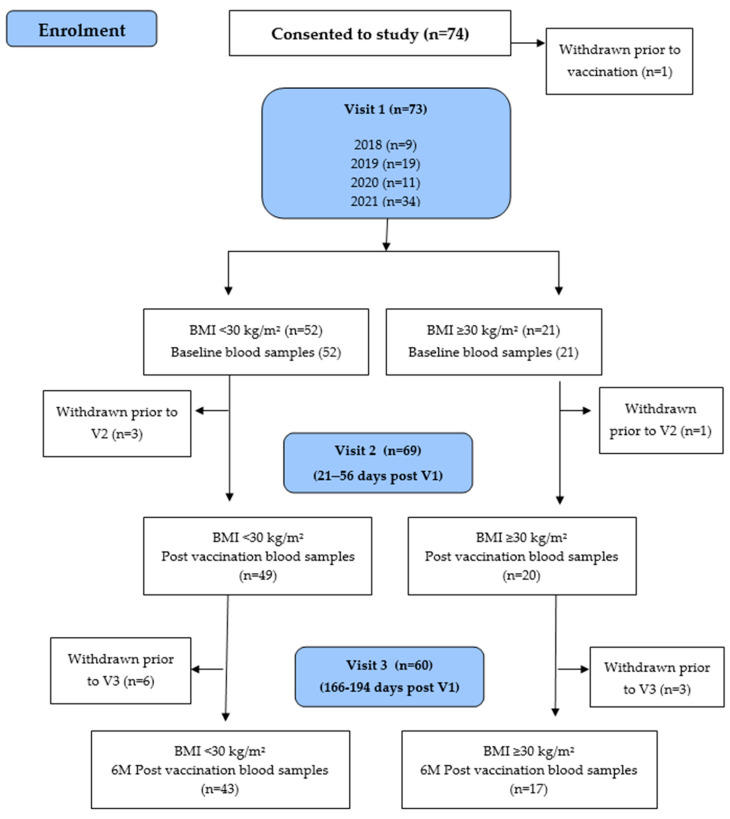
Participant flow chart.

Whilst the study aimed to enrol 150 participants, institutional rules during the COVID-19 pandemic impacted study recruitment, impacting the overall power of the study. The COVID-19 pandemic also affected retention of study participation through to study completion. Enrolled participants had a blood sample collected at baseline (prior to vaccine administration) at 1 month following vaccination and again at approximately 6–7 months following vaccination (post-delivery). In addition, cord blood was collected (where possible) at the time of delivery to measure antibody responses to the vaccines administered. Pre-vaccination blood samples were collected from 73 enrolled participants, of whom 21 (29%) were obese. Post-vaccination blood samples were collected from 69 participants at 1 month post-vaccination and at 60 participants at 6 months post-vaccination. Cord blood samples were collected from 52 (71%) participants at delivery (see Figure 1, Table 1).

### 2.2. Serology and Immunogenicity Assessment

Serum samples were sent to the WHO Collaborating Centre for Reference and Research on Influenza (Melbourne, Australia) to measure influenza antibody titres by hemagglutination inhibition (HI) assay according to the WHO method [24]. HI antibody titrations of the participant serum samples were all run in the same assay for each virus tested to reduce assay variability. Any sera featuring titres ≥ 1280 were re-titrated to obtain an endpoint titre. HI results that were negative (i.e., agglutination apparent at <1:10 dilution) were assigned a titre of 5 for subsequent calculations. Post-infection ferret antisera raised to the appropriate wild-type virus for each type/subtype/lineage vaccine virus tested were used as positive controls in all assays and compared with previously obtained titres. Viruses used for HI analysis were matched to the vaccines received and included A/H1N1, A/H3N2, B/Victoria and B/Yamagata viruses. Additionally, 1% (*v*/*v*) Turkey red blood cells were used in all HI assays. HI titres ≥ 40 were considered seroprotective (SP).

### 2.3. Design and Statistical Analysis

The primary aim of the study was to compare the proportion achieving seroprotection in women with pregnancy BMI < 30 kg/m^2^ to those with BMI ≥ 30 kg/m^2^) to determine whether the proportions were different between the groups. Assuming a 1:2 ratio of obese to non-obese participants (based on population obesity characteristics), and with the assumption that the proportion of non-obese participants who fail to achieve seroprotective titres to at least 1 strain following influenza vaccination is 0.2 (20%), if the true failure rate for obese participants is 0.5 (50%), 33 obese and 66 non-obese participants were required to be able to reject the null hypothesis with 80% power.

The proportions of obese vs. non-obese pregnant women in the population with HI titres ≥ 40 units were compared using a z-test of two proportions for each influenza strain individually and for the composite of all four influenza vaccine strains following influenza vaccination. Mann–Whitney tests were used to compare Geometric Mean Antibody Titres (GMTs) and GMT Ratios (GMTRs) between obese and non-obese groups at each timepoint. A multivariable log binomial model was used to estimate the risk of seroprotection against all four strains for obese compared to non-obese pregnant women at 1 and 6 months post-vaccination and at delivery. The models were adjusted for the trimester during which vaccination was given and for coadministration of a pertussis vaccine. Variables for inclusion in the multivariable model were prespecified and selected for their potential impact on the outcome, recognising the limitations brought about by the sample size. Statistical significance was assessed at the 0.05 level using two-sided tests. Effect estimates are presented with their 95% confidence intervals.

This study was approved by the Women’s and Children’s Health Network Human Research Ethics Committee (HREC/17/WCHN/63) and registered at Clinicaltrials.gov (NCT03457194) prior to recruitment.

## 3. Results

### 3.1. Participants

A total of 74 pregnant women consented to participating in the study, with one participant withdrawing prior to vaccination. Of the 73 participants who were consented and vaccinated, 52 (71%) had a pre-vaccination BMI < 30 kg/m^2^ and 21 (29%) had a pre-vaccination BMI ≥ 30 kg/m^2^ (Figure 1). Otherwise, the demographic characteristics and vaccination histories were similar between groups There were no underweight participants (BMI < 18 kg/m^2^) included in the study. There were 26 women with a BMI of between 18 and 24.9 kg/m^2^ and 26 women with a BMI of between 25 and 29 kg/m^2^. For the obese group, 13 had a BMI of between 30 and 34.9 kg/m^2^, four women had a BMI of between 35 and 39.9 kg/m^2^ and four women had a BMI greater than 40 kg/m^2^. The highest BMI was 43.7 kg/m^2^.

The mean age of enrolled participants was 32.5 years (SD 4.6 years; range 21–44 years). The mean gestation at vaccination was 23.6 weeks (SD 7.1 weeks, range 11–37 weeks). Most participants were born in Australia (71%) and had previously received an influenza vaccine (74%). The mean interval between vaccination and sample collection at each timepoint was similar for both groups (Table 1).

### 3.2. Vaccine Responses: Geometric Mean Titres

The pre-vaccination Geometric Mean HI antibody titres were similar for obese vs. non-pregnant women for both influenza A/H3N2 and influenza A/H1N1; however, obese pregnant women had lower mean influenza B GMTs prior to vaccination, particularly for B/Victoria (mean obese 27.8, 95% CI 17.9–43.3 vs. mean non-obese 49.5, 95% CI 38.7–63.3, *p* = 0.03). At 1 month post-vaccination, both obese and non-obese pregnant women groups demonstrated 2–10-fold increases in GMTs for each of the four influenza strains (A/H3N2, A/H1N1, B/Yamagata and B/Victoria), with the highest rise in GMTs being evident for A/H1N1 (an approximately 10-fold increase from baseline for the obese group and a 6-fold increase for the non-obese group).

**Figure 2:**
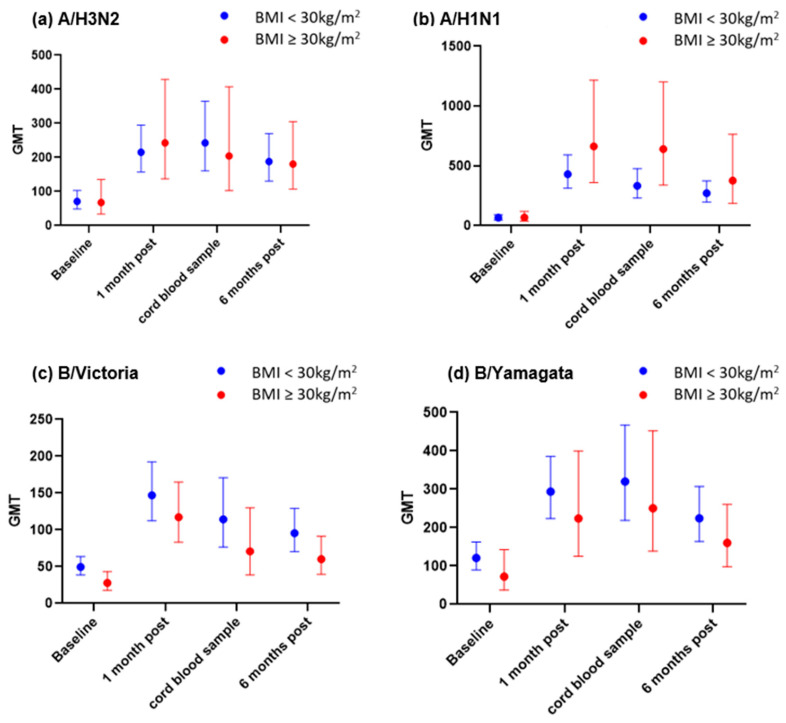
Pre- and post-vaccination HI titres by BMI category for (**a**) A/H3N2; (**b**) A/H1N1; (**c**) B/Victoria; and (**d**) B/Yamagata.

Whilst antibody titres declined by 6 months post-vaccination, they remained above pre-vaccination levels for all four influenza antigens. Cord blood samples also demonstrated elevated HI antibody titres for all influenza antigens for both obese and non-obese pregnant women compared with baseline antibody titres (Table 2, Figure 2).

### 3.3. Impact of Coadministration of Adacel

Almost half of the participants had Adacel coadministered at the time of their influenza vaccination (32/73, 44%) (Table 1). There were no significant differences in the Geometric Mean HI Titres to any of the four influenza vaccine strains at any of the post-vaccination timepoints (1 m, at delivery and at 6 m) for women who received a FluQuadri vaccine alone or who were coadministered with Adacel (Supplementary Table S1).

### 3.4. Seroprotection

For influenza vaccines, one evaluation criterion used to assess vaccine immunogenicity is the proportion of recipients who achieved a HI titre of ≥40 at 1 month post-vaccination [25]. This threshold is estimated to confer approximately 50–70% protective efficacy against clinical symptoms of infection. Over three-quarters of the non-obese pregnant women had antibody titres ≥ 40 to all four influenza vaccine strains prior to receiving the vaccine. Similar proportions were evident for the obese vs. non-obese pregnant women for A/H3 (71% vs. 75%, *p* = 0.75), and A/H1 (81% vs. 75%, *p* = 0.59); however, obese pregnant women appeared to have slightly lower pre-vaccination titres as compared to non-obese pregnant women for B/Yamagata (76% vs. 92%, *p* = 0.06) and B/Victoria (48% vs. 79%, *p* < 0.01) (Table 2).

At 1 month post-vaccination, the proportion with seroprotective antibody titres was greater than 95% for both obese and non-obese groups for each of the four influenza strains. Seroprotective antibodies were sustained over 6 months for both obese and non-obese groups for both the influenza A/H3N2 (94% vs. 95%) and A/H1N1 (100% vs. 100%) strains. A high proportion of cord blood samples from both obese and non-obese pregnant women also demonstrated seroprotective antibody titres in regard to A/H3N2 (88% vs. 94%) and A/H1N1 (100% vs. 100%). For influenza B strains, however, although the proportion of samples with seroprotective antibody titres remained high, it was lower for the obese group compared to the non-obese group for both the cord blood samples (B/Vic: 76% vs. 91%, *p* = 0.14; B/Yam: 94% vs. 100%, *p* = 0.15) and the 6-month-post-vaccination samples (B/Vic: 82% vs. 88%, *p* = 0.54; B/Yam:88% vs. 100%, *p* = 0.02).

### 3.5. Predictors of Optimal Seroprotective Responses

Optimal seroprotective response was defined as a HI Titre ≥ 40 for all four influenza strains (A/H3N2, A/H1N1, B/Yamagata and B/Victoria).

Almost half of the enrolled participants (34/73; 22%) had pre-vaccination HI titres ≥ 40 for all four of the influenza strains tested (A/H1N1, A/H3N2, B/Yamagata and B/Victoria). At 1 month post-vaccination, this proportion increased to 96% (n = 66/69) of participants with seroprotective antibody titres to all four vaccine component antigens, indicating robust immediate vaccine responses regardless of BMI group (Table 3). At 6 months post-vaccination, and for cord blood samples, a lower proportion had HI titres ≥ 40 for all four influenza vaccine strains for the obese vs. non-obese group (6 m: 71% vs. 84%, *p* = 0.32; cord: 65% vs. 86%, *p* = 0.14). In the multivariable log-binomial models adjusting for trimester at vaccination and coadministration of a pertussis vaccine, obese pregnant women had a 25% lower risk regarding maintaining seroprotective antibody titres through to 6 months post-vaccination for all four influenza strains compared to non-obese women (aRR 0.75, 0.53–1.08, *p* = 0.13). Similarly, obese pregnant women had a 25% lower risk for seroprotective antibody titres for all four influenza strains in their cord blood samples compared to non-obese pregnant women (aRR 0.75, 0.54–1.05, *p* = 0.10) (Table 3).

### 3.6. Safety

There were 15 serious adverse events (SAEs) reported for 10 maternal participants during their study participation. All SAEs were deemed unrelated to the study vaccines following review and investigation by a study medical officer. SAEs were reported for 9/52 participants in the non-obese group (17%) vs. 1/21 (5%) in the obese group (*p* = 0.26).

## 4. Discussion

Both obese and non-obese pregnant women achieved robust antibody responses to all influenza vaccine strains at 1 month post-vaccination, with 95–100% of vaccine recipients achieving HI titres of ≥40. However, our study suggests that obese pregnant women were less likely to maintain seroprotective antibody levels at the 6-month-post-vaccination sample timepoint in regard to all four influenza vaccine antigens compared with non-obese pregnant women. Similarly, cord blood samples collected from obese pregnant women were less likely to have seroprotective antibody levels against all four influenza vaccine antigens compared with cord blood samples from non-obese pregnant women.

For influenza A, almost all participants achieved, maintained to 6 months, and adequately transferred antibodies to their infant regardless of BMI category. For influenza B lineage strains, whilst the obese group had lower GMTs for both B/Yamagata and B/Victoria lineage antibodies than the non-obese group across all timepoints (baseline, 1 m, cord and 6 m), the proportion achieving seroprotective antibody titres (HI ≥ 40) at 1 m, cord sample and 6 m post-vaccination remained above 75%. For all influenza strains, and at each sample timepoint, the proportion of participants with seroprotective HI titres remained above the 70% threshold specified by the European Agency for the Evaluation of Medicinal Products (EMEA) as one of the criteria for serological assessment of influenza vaccine response that was previously used by the agency [25]. A serum HI antibody titre ≥40 has been considered to be associated with >50% reduction in the risk of subsequent influenza disease or infection [26,27,28].

Our study results support the findings of other published studies which indicate that obesity does not significantly impair the ability to achieve seroprotective antibody responses following influenza vaccination in pregnant [20,21] or non-pregnant adults [29,30,31] at 1 month post-vaccination, adding the additional information that these robust antibody responses are well maintained through to 6 months post-vaccination and are adequately transferred to the foetus.

GMTs and seroprotective antibody levels were lower for influenza B strains at baseline timepoints for obese vs. non-obese pregnant women, which may suggest more rapid waning of previously acquired antibodies in obese vs. non-obese groups, but this was not observed for influenza A strains. This may be due to the higher frequency of repeat exposure to circulating influenza A viruses in the community compared with influenza B strains allowing for the natural boosting of antibodies to mask any impact of the more rapid waning of antibodies in obese persons. A study from Sheridan et al. assessing obesity and influenza vaccine responses in a non-pregnant adult population showed that obese populations had a more rapid waning of vaccine-induced antibodies at 12 months post-vaccination compared to non-obese vaccine recipients [17], and it also demonstrated that T-cell activation was more impaired in obese individuals compared with healthy weight individuals. Evidence for an association between obesity and the more rapid waning of vaccine responses has also been shown for COVID-19 vaccines [32,33], with authors suggesting that increased glycosylation of antibodies or a lack of high-affinity antibodies may be implicated in the impaired longevity of vaccine antibody responses in obese persons.

Whilst our data showed adequate seroprotective titres amongst obese and non-obese pregnant women at each timepoint, there was some evidence that antibodies waned more quickly in obese vs. non-obese participants for some influenza strains, although this was not demonstrable as being statistically significant with our sample size. This finding may have implications for obese women vaccinated in early pregnancy. In Australia, influenza vaccination is recommended at any stage of pregnancy, and a recent cohort study found that 25% of women were vaccinated in their first trimester [34]. We have also previously shown that influenza B strains may cause more severe illness in young children [35]; therefore, determining optimal maternal vaccination timing to provide adequate protection against both influenza A and influenza B is an important public health strategy. Recent surveillance, however, suggests that B/Yamagata lineage influenza strains have disappeared globally since the COVID-19 pandemic [36]. The World Health Organisation has therefore advised that the B/Yamagata lineage antigen is no longer warranted for inclusion in 2024–2025 influenza vaccines, but the B/Victoria lineage viruses have continued to circulate [37].

This study does have several limitations. Firstly, the reduced sample size achieved relative to the planned sample size restricted the variables that could be considered in adjusted models; therefore, the study was underpowered in regard to detecting the anticipated differences between groups. Small but clinically important differences between obese and non-obese groups may be difficult to demonstrate without a larger sample size. In addition, the participants enrolled were not a true random sample of obese and non-obese pregnant women. Given these limitations, the study findings may not be generalisable to the wider population of pregnant women and larger studies would be required to provide further evidence regarding the role of obesity in vaccine antibody longevity.

Whilst obesity is one factor that has been implicated in impaired immunogenicity, many other host and environmental factors may also impact the immunogenicity of influenza vaccines [38,39]. While our regression models adjusted for trimester at vaccination and coadministration of pertussis vaccination, other important potential confounding variables, such as vaccination history, other health conditions, microbiome and genetics, may also influence vaccine responses and were not assessed or controlled for in our study. Furthermore, more than 75% of our participants had received a prior influenza vaccine and had seroprotective titres at baseline. Therefore, our study findings may not be generalisable to vaccine-naïve populations.

Additionally, this study only assessed humoral immunity and did not measure potential differences in cell-mediated immunity that may play an important role in protection against influenza. It has been increasingly recognised that, whilst the HI titre ≥ 40 threshold has been the most commonly accepted correlate for protection, it may not apply equally to all groups and other measures of protection are also important to consider [40,41]. Finally, it is important to note that equivalent HI titres may not translate to equivalent protection against influenza illness for groups with and without obesity. A large prospective observational study in adults who received trivalent influenza vaccine showed that vaccinated obese participants had twice the risk of influenza or influenza-like illness compared with vaccinated healthy weight participants despite similar immunogenicity [42]. Therefore, it is important to continue to monitor vaccine efficacy to ensure that current influenza vaccine programmes and policies adequately protect obese and non-obese pregnant women and their newborns. Obese pregnant women vaccinated in early pregnancy may benefit from a second influenza vaccination during pregnancy to ensure optimal seroprotection against influenza for their newborns; however, larger studies are required to evaluate this. Additional studies assessing immunogenicity at 12 months post-vaccination in obese vs. non-obese individuals would be useful to determine whether twice yearly influenza vaccinations may provide additional benefits for people living with obesity.

## 5. Conclusions

In conclusion, our study was able to demonstrate that, whilst obesity did not impact the attainment of seroprotective antibody titres at 1 month post-vaccination, there was an indication that the maintenance of antibodies for 6 months and placental transfer of influenza antibodies may be lower for obese compared to non-obese pregnant women for some influenza strains. Future studies with a larger cohort are required to understand whether the current vaccine policy is adequate at a population level for protecting obese pregnant women and their infants.

## Figures and Tables

**Table 1:** Participant summary and characteristics.

Characteristic	BMI < 30 kg/m^2^	BMI ≥ 30 kg/m^2^
	n = 52	n = 21
Maternal age at vaccination mean years, SD [range]	32.8 (4.6) [24–44]	31.7 (4.5) [21–39]
Maternal BMI (first trimester) mean kg/m^2^, (SD) [range]	25.7 (2.5) [19.4–29.9]	34.3 (4.6) [30.1–43.7]
Country of birth (Australia) (%) yes	37/52 (71%)	15/21 (71%)
Maternal gestation at vaccination mean completed weeks, (SD) [range]	23.6 (7.4) [11–37]	23.7 (6.8) [13–33]
Prior influenza vaccination (ever) (%) yes	39/52 (75%)	15/21 (71%)
Pertussis vaccine coadministered (%) yes	21/52 (40%)	11/21 (52%)
Infant gestation at delivery mean completed weeks, SD [range]	38.4 (1.8) [31–42]	38.6 (1.0) [37–41]
Infant Sex (Male: Female: Unknown)	25:24:3	12:8:1
Interval between vaccination and blood sample:		
1 m post-vaccination: Mean days (SD) [range]	31 (8.1) [21–56]	31 (7.5) [21–47]
6 m post-vaccination: Mean days (SD) [range]	180 (13.6) [166–222]	184 (16.7) [166–227]
Cord blood: Mean days post-vaccination (SD) [range]	106 (51.8) [13–196]	105 (53.5) [39–193]

**Table 2:** Immune responses to influenza vaccine in pregnant women by influenza type/lineage and BMI category at each sample timepoint.

		A/H3N2			A/H1N1			B/Victoria			B/Yamagata		
		BMI < 30 kg/m^2^	BMI ≥ 30 kg/m^2^	*p* Value	BMI < 30 kg/m^2^	BMI ≥ 30 kg/m^2^	*p* Value	BMI < 30 kg/m^2^	BMI ≥ 30 kg/m^2^	*p* Value	BMI < 30 kg/m^2^	BMI ≥ 30 kg/m^2^	*p* Value
GMT (95% CI)	Pre-vaccination	71.0 (48.8–103.1)	67.8 (34.0–135.3)	0.98	66.4 (48.2–91.4)	67.8 (38.6–119.2)	0.93	49.5 (38.7–63.3)	27.8 (17.9–43.3)	0.03	120.9 (89.9–162.7)	72.5 (36.8–142.9)	0.34
	1 month post	215.3 (157.1–295.0)	242.5 (137.1–429.1)	0.92	430.7 (313.6–591.6)	662.6 (360.4–1218.1)	0.31	147.0 (112.4–192.2)	117.1 (83.3–164.7)	0.41	294.0 (224.1–385.7)	223.8 (125.3–399.6)	0.58
	GMTR (1 m/pre)	3.01 (2.26–4.01)	3.86 (2.06–7.23)	0.56	6.37 (4.17–9.76)	10.93 (5.09–23.42)	0.19	3.01 (2.29–3.97)	4.43 (2.76–7.13)	0.19	2.43 (1.83–3.25)	3.32 (1.47–7.54)	0.58
	6 months post	188.0 (130.8–270.3)	180.8 (107.1–305.1)	0.85	272.4 (198.8–373.3)	376.7 (185.7–764.3)	0.43	95.5 (70.6–129.2)	60.1 (39.6–91.4)	0.09	224.5 (164.0–307.1)	160.0 (98.2–260.7)	0.66
	GMTR (6 m/1 m)	0.75 (0.63–0.89)	0.59 (0.43–0.79)	0.20	0.64 (0.53–0.76)	0.57 (0.37–0.86)	0.65	0.68 (0.57–0.81)	0.54 (0.38–0.77)	0.24	0.76 (0.65–0.89)	0.76 (0.50–1.16)	0.63
	Cord blood	242.51 (61.0–365.1)	204.4 (102.6–407.2)	0.61	332.9 (232.2–477.4)	640 (340.9–1201.7)	0.12	114.3 (76.5–170.7)	70.8 (38.6–129.9)	0.28	320 (219.1–467.3)	250.6 (138.9–452.1)	0.67
	GMTR (cord/1 m)	1.00 (0.78–1.28)	0.78 (0.52–1.19)	0.28	0.92 (0.74–1.18)	0.92 (0.60–1.42)	0.84	0.77 (0.63–0.94)	0.64 (0.43–0.95)	0.60	1.04 (0.87–1.25)	1.06 (0.65–1.17)	0.71
% HI ≥ 40	Pre-vaccination	39/52 (75%)	15/21 (71%)	0.75	39/52 (75%)	17/21 (81%)	0.59	41/52 (79%)	10/21 (48%)	<0.01	48/52 (92%)	16/21 (76%)	0.06
	1 month post	47/49 (96%)	20/20 (100%)	0.36	49/49 (100%)	20/20 (100%)	-	49/49 (100%)	20/20 (100%)	-	49/49 (100%)	19/20 (95%)	0.11
	6 months post	41/43 (95%)	16/17 (94%)	0.84	42/43 (98%)	17/17 (100%)	0.53	38/43 (88%)	14/17 (82%)	0.54	43/43 (100%)	15/17 (88%)	0.02
	Cord blood	33/35 (94%)	15/17 (88%)	0.44	35/35 (100%)	17/17 (100%)	-	32/35 (91%)	13/17 (76%)	0.14	35/35 (100%)	16/17 (94%)	0.15

**Table 3:** Univariate and multivariable binomial regression models for association between BMI categories and seroprotection (HI Titre ≥ 40 for all four influenza strains at each sample timepoint).

Timepoint	HI ≥ 40 for All 4 Strains		RR (95% CI)	*p* Value	aRR * (95% CI)	*p* Value
	BMI < 30 kg/m^2^	BMI ≥ 30 kg/m^2^				
Pre-vaccination	27/52 (52%)	7/21 (33%)	0.64 (0.33–1.24)	0.19		
1 month post	47/49 (96%)	19/20 (95%)	0.99 (0.88–1.11)	0.87	0.94 (0.83–1.06)	0.30
6 months post	36/43 (84%)	12/17 (71%)	0.84 (0.60–1.18)	0.32	0.75 (0.53–1.08)	0.13
Cord blood	30/35 (86%)	11/17 (65%)	0.75 (0.52–1.10)	0.14	0.75 (0.54–1.05)	0.10

* Post-vaccination timepoints are adjusted for trimester at vaccination and coadministration of pertussis.

## Data Availability

De-identified data presented in this study are available on request from the corresponding author.

## Supplementary Materials

The following supporting information can be downloaded at: https://www.mdpi.com/article/10.3390/vaccines12121307/s1, Table S1: Antibody responses to influenza vaccine in pregnant women by vaccines received (FluQuadri alone vs FluQuadri + Adacel).
