# Supplementary material for: The Impact of Obesity on Influenza Vaccine Immunogenicity and Antibody Transfer to the Infant During Pregnancy
Source: Vaccines (Basel). 2024 Nov 22;12(12):1307. doi: 10.3390/vaccines12121307 (PMC11680122; doi:10.3390/vaccines12121307)
Supplement: Supplementary file 1 [file vaccines-12-01307-s001.zip › vaccines-3286268-supplementary.pdf]

Table S1: Antibody responses to influenza vaccine in pregnant women by vaccines received (FluQuadri alone vs FluQuadri + Adacel).

	**Time-point**	**Fluquadri only (n=41)**	**Fluquadri +Adacel (n=32)**	
		**GMT (95%CI)**	**GMT**	**p value**
**A/H3N2**	Pre-vaccination	90.1 (62.1-130.7)	50.8 (28.8-89.4)	0.08
	1 month post	245.1 (181.0-332.0)	197.0 (119.5-324.8)	0.67
	6 months post	176.7 (122.5-254.7)	199.7 (120.3-331.7)	0.65
	Cord blood	240.2 (162.8-354.5)	216.3 (114.3-409.3)	0.96
**A/H1N1**	Pre-vaccination	72.3 (55.4-94.3)	60.4 (35.3-103.4)	0.53
	1 month post	490.2 (320.9-749.0)	485.0 (335.1-702.1)	0.79
	6 months post	289.8 (195.0-430.9)	311.3 (195.8-494.7)	0.48
	Cord blood	387.4 (240.4-624.5)	445.8 (289.4-686.8)	0.46
**B/Vic**	Pre-vaccination	40.0 (30.1-53.2)	44.6 (31.1-63.9)	0.81
	1 month post	124.7 (95.2-163.5)	156.4 (110.2-221.8)	0.32
	6 months post	73.9 (52.1-104.9)	99.9 (70.5-141.4)	0.16
	Cord blood	76.3 (49.4-117.7)	133.5 (80.0-223.0)	0.06
**B/Yam**	Pre-vaccination	106.6 (75.2-151.2)	101.5 (62.2-165.7)	0.91
	1 month post	248.1 (176.0-349.8)	305.6 (209.4-445.9)	0.29
	6 months post	195.0 (133.8-284.3)	217.1 (151.3-311.4)	0.42
	Cord blood	290.8 (195.6-432.5)	301.3 (178.0-510.0)	0.92
